# Altered Inhibitory Control Across Subclinical Depression and Major Depressive Disorder: Evidence From Stroop Tasks and Diffusion Modeling

**DOI:** 10.1155/da/5711923

**Published:** 2026-10-25

**Authors:** Qingying Ye, Yi-shuo Qiao, Jia-jia Jin, Si-yu Zhang, Yun-da Li, Wen-jie Dou, Kai Jin, Ke Ni, Zhong Yang, Bao-ming Li, Xin-lu Cai

**Affiliations:** ^1^ Zhejiang Philosophy and Social Science Laboratory for Research in Early Development and Childcare Hangzhou Normal University Hangzhou China hznu.edu.cn; ^2^ Institute of Brain Science School of Basic Medical Sciences Hangzhou Normal University Hangzhou China hznu.edu.cn; ^3^ Turku PET Centre Turku University Hospital and University of Turku Turku Finland utu.fi; ^4^ Department of Psychology Jing Hengyi School of Education Hangzhou Normal University Hangzhou China hznu.edu.cn; ^5^ Qiqihar Mental Health Center Qiqihar China; ^6^ The Fifth People’s Hospital of Lin’an District Hangzhou China

**Keywords:** depression, drift diffusion model, inhibitory control, Stroop task, subclinical depression

## Abstract

Inhibitory control deficits are central to cognitive dysfunction in major depressive disorder (MDD), yet how such deficits vary across subclinical depression and MDD remains unclear. Childhood trauma may further contribute to variability in inhibitory control. This study examined behavioral and computational markers of inhibitory control across nonemotional and emotional contexts in healthy, subclinical depression, and MDD groups and explored associations with childhood trauma. Thirty individuals with subclinical depression, 33 patients with MDD, and 36 healthy controls (HCs) completed three Stroop tasks (color‐word, emotion, and face‐word), during which reaction times (RTs) were recorded. Latent cognitive parameters were estimated using the drift diffusion model (DDM) and diffusion model for conflict tasks (DMCs). Associations between these indicators and childhood trauma were examined. Patients with MDD showed significantly longer RTs than subclinical individuals in emotion and face‐word Stroop tasks. Computational modeling revealed prolonged nondecision time in MDD, relative to subclinical depression (color‐word Stroop task) and HCs (emotion Stroop task). In the subclinical group, greater childhood trauma was associated with faster RTs in incongruent face‐word trials and shorter nondecision time in the color‐word Stroop task. Our findings support a possible inverted U‐shaped pattern of inhibitory control across depressive severity, with enhanced performance in subclinical depression but poorer performance in MDD. Enhanced inhibitory control in subclinical depression may reflect compensatory adaptation linked to early adversity. These findings provide novel insights into stage‐related differences of inhibitory control in depression.

## 1. Introduction

Major depressive disorder (MDD) is a common and disabling mental disorder characterized by impairments in cognitive control, which contribute to attentional biases [1], dysregulated emotion processing [2], impaired decision‐making [3], and increased risk for suicidal ideation and behaviors [4]. A comparable but less severe pattern of depressive symptoms is observed in subclinical/subthreshold depression, which does not meet the diagnostic criteria for MDD [5]. Subclinical depression is more prevalent in the general population and has been identified as a precursor and risk factor for MDD [6]. According to cognitive theories of depression [2, 7], deficits in cognitive control play a key role in the onset, development, and maintenance of depressive symptoms. Investigating cognitive control dysfunction in both subclinical depression and MDD may therefore provide critical insight into the psychopathology of depression and advance the development of preventive and therapeutic interventions.

Inhibition control, a core component of cognitive control, refers to the ability to suppress interference from irrelevant stimuli [8]. To examine inhibitory control in response to nonemotional and emotional distractions, previous studies have employed the color‐word Stroop task and the emotion Stroop task, respectively [9–11]. In the color‐word Stroop task, incongruent trials elicit longer reaction times (RTs) due to inference from the dominant word‐reading process [12]. Similarly, the emotion Stroop task uses affectively valenced words instead of color terms, with emotionally salient words generally leading to delayed RTs than neutral words [13–15]. A meta‐analysis study reported that patients with depression exhibited slower color‐naming performance than healthy controls (HCs) in both tasks, with greater depression severity associated with larger effect sizes [16]. The face‐word Stroop task was later developed to assess emotional conflict processing by generating interference between emotional facial expressions (i.e., target) and incongruent emotional word labels (i.e., distractor) [17, 18]. Patients with MDD and individuals with subclinical depression both showed delayed response in this task compared to HCs [19–21], indicating difficulties in resolving emotional conflict.

Interestingly, some studies suggest an inverted U‐shaped relationship between depression severity and cognitive control: Mild to moderate depressive symptoms may enhance cognitive control, whereas more severe symptoms impair it [22, 23]. For example, Zhou et al. [24] reported a compensatory inhibitory control in individuals with subsyndromal depression during the go/no‐go task, evidenced by decreased N2 amplitudes and increased P3 amplitudes in the no‐go condition compared to HCs. Despite these previous findings, the evidence remains inconsistent, particularly when comparing subclinical and clinical populations. A comprehensive investigation using the multiple Stroop tasks mentioned above in both subclinical depression and MDD may help elucidate the cognitive underpinnings of depressive symptoms.

Importantly, observed behavioral impairments may not only reflect inhibitory dysfunction but also latent cognitive processes, such as processing efficiency, response caution, stimuli encoding, and motor actions [25]. To decompose these components, recent studies have employed the drift diffusion model (DDM) and its variant, the diffusion model for conflict task (DMC), to explore the cognitive mechanisms underlying Stroop task performance [26–28]. Compared to traditional analyses of RT and accuracy, the DDM and the DMC are more sensitive to trial‐by‐trial intraindividual variability and provide a more nuanced understanding of the cognitive processes underlying decision‐making [25, 29]. Using various behavioral paradigms, empirical studies using the DDM have demonstrated less efficiency of evidence accumulation, greater caution or uncertainty to make decisions, and impaired sensorimotor response in patients with depression [3, 30], individuals with subclinical depression [31], and healthy individuals with depressive symptoms [32]. However, the specific latent cognitive components contributing to performance impairments during Stroop tasks in subclinical depression and MDD remain unclear.

Additionally, childhood trauma, identified as a vulnerability factor for depression, has been linked to cognitive control deficits [33]. For example, a recent study reported that a history of emotional abuse (EA) during childhood in patients with MDD was associated with hypoactivity in brain regions of the cognitive control network [34]. Alternatively, enhanced cognitive control may act as a protective factor, facilitating adaptation to chronic stress induced by childhood trauma [35]. Indeed, one study found that EA was related to better performance of executive function in patients with MDD [36]. Therefore, childhood trauma was included as an exploratory individual difference factor to examine whether early adversity is associated with variability in inhibitory control within different depression stages.

The present study aimed to investigate inhibitory control performance in both subclinical depression and MDD using three Stroop tasks (color‐word, emotion, and face‐word). To elucidate the underlying cognitive mechanisms, we employed computational modeling approach (the DDM and the DMC) to estimate and compare the decision‐making parameters across groups. We further aimed to explore how childhood trauma was related to the performance of the Stroop tasks. Based on prior research [22, 23, 37, 38], we hypothesized that, compared to HCs, patients with MDD would exhibit slower RTs, whereas individuals with subclinical depression would show faster performance, potentially reflecting compensatory mechanisms. We expected similar patterns to emerge in the DDM and the DMC parameters. Furthermore, we hypothesized that childhood trauma would be associated with poorer performance in patients with MDD but enhanced or compensatory performance in individuals with subclinical depression.

## 2. Methods

### 2.1. Participants

Our sample consisted of patients with MDD, individuals with subclinical depression, and HCs. Thirty‐three patients with MDD were recruited from the Fifth People’s Hospital of Lin’an District in Zhejiang province and Qiqihar Mental Health Center in Heilongjiang province. All MDD diagnoses were made by experienced psychiatrists according to the Diagnostic and Statistical Manual of Mental Disorders, Fifth Edition (DSM‐5) [39]. The 24‐item Hamilton [40] Rating Scale for Depression (HAMD‐24) was used to assess clinician‐rated depressive symptom severity in the MDD group. The mean illness duration was 4.51 years (SD = 3.10), the average dosage of the antidepressant was 9.64 mg/day (fluoxetine equivalence, SD = 15.01), and the mean score of HAMD was 20.30 (SD = 6.89). In addition, 30 individuals with subclinical depression and 36 HCs were recruited from a large cohort of college students. In addition, all participants completed the Beck Depression Inventory (BDI) [41], which provided a common self‐report measure of depressive symptoms across the three groups. Based on the scores of the BDI [42, 43], participants scoring above 16 were categorized as individuals with subclinical depression, while those scoring lower than 16 were categorized as HCs. To ensure the absence of potential psychiatric disorders, both individuals with subclinical depression and HCs were screened using the Mini International Neuropsychiatric Interview (MINI) [44].

Exclusion criteria for all participants included an estimated IQ below 70, any history of any neurological disorder, alcohol or drug dependence, or severe head injury. Patients with comorbid psychiatric disorders were also excluded. Additionally, patients with MDD should not have a history of transcranial magnetic stimulation or electroconvulsive therapy in the past 3 months. All participants had normal or corrected‐to‐normal vision, normal color vision, and were right‐handed. Participants whose task response accuracy fell below chance level (50%) were excluded [26]. This study was approved by the Ethics Committees of the School of Basic Medical Sciences, Hangzhou Normal University (Protocol Number 20221206). All participants gave written informed consent.

### 2.2. Stroop Tasks

Adapted from the paradigms developed by Huang et al. [14], the present study included three Stroop tasks: the color‐word Stroop task, the emotion Stroop task, and the face‐word Stroop task (Figure 1). Participants were instructed to respond as quickly and accurately as possible during each Stroop task.

**Figure 1:**
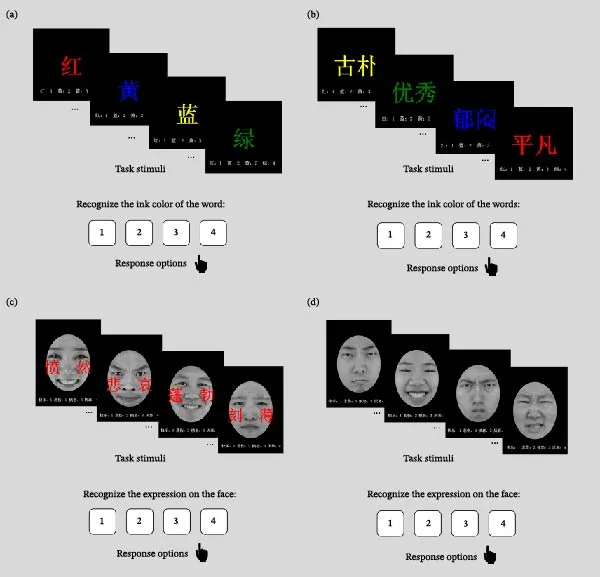
Experimental tasks. (a) Color‐word Stroop task. (b) Emotion Stroop task. (c) Face‐word Stroop task. (d) Emotion recognition task.

In the color‐word Stroop task, participants were presented with four Chinese color characters: “red,” “blue,” “yellow,” and “green.” Each character was displayed in one of four corresponding ink colors: red (RGB: 255, 0, 0), blue (RGB: 0, 0, 255), yellow (RGB: 255, 255, 0), or green (RGB: 0, 128, 0), resulting in both congruent (e.g., “red” in red ink) and incongruent (e.g., “red” in blue ink) conditions. Participants were asked to identify the ink color of each character while ignoring its semantic content. Responses were made with the dominant hand by pressing keys on a keyboard: “1” for red, “2” for blue, “3” for yellow, and “4” for green. Longer RTs in the incongruent condition reflect increased cognitive interference due to the semantic‐color conflict.

The emotion Stroop task followed the same procedure as the color‐word Stroop task, except that the stimuli consisted of emotionally valenced (positive and negative) and neutral words instead of color characters. Participants were required to identify the ink color of the words while ignoring their meanings. Negative emotional words were expected to elicit greater interference effects [11].

In the face‐word Stroop task, participants were presented with four types of emotional facial expressions (happy, sad, angry, and disgust). An emotional word corresponding to one of these emotions was displayed on both cheeks of each face. The word either matched (congruent condition) or mismatched (incongruent condition) the facial expression. Participants were instructed to identify the emotion expressed by the face while ignoring the emotional word. Emotional cognitive conflict arises when the distractor words activate a competing affective response, engaging overlapping neural systems [18].

The words and facial stimuli were selected from the Chinese affective word system [45] and the Tsinghua facial expression database [46], respectively. More detailed information of the tasks can be found in the Supporting Information.

### 2.3. Other Assessment

Following the Stroop tasks, participants completed an emotion recognition task to assess whether the three groups had comparable abilities in recognizing four types of facial expressions. Response keys were consistent across tasks involving facial stimuli, with “1” for happy, “2” for sad, “3” for angry, and “4” for disgust.

The Chinese version of the Childhood Trauma Questionnaire‐Short Form (CTQ‐SF) is a 28‐item questionnaire for assessing early traumatic experiences [47, 48]. It measures five types of childhood trauma: emotional neglect (EN), EA, physical neglect (PN), physical abuse (PA), and sexual abuse (SA). Each item is rated on a five‐point Likert scale, with higher scores indicating more severe childhood trauma. The Cronbach’s alpha coefficients for the subscales of the CTQ‐SF in the present study ranged from 0.62 to 0.81 (EN: 0.77; EA: 0.70; PN: 0.62; PA: 0.70; and SA: 0.81).

The short form of the Chinese version of the Wechsler Adult Intelligence Scale‐Revised (WAIS‐R; information, arithmetic, similarities, and digit span subtests) was used to estimate participants’ IQ [49].

### 2.4. Procedure

At the beginning of the session, the experimental procedure was explained to the participants in detail. Then, they completed the self‐report questionnaire. Next, participants were instructed to complete the three Stroop tasks. To ensure that participants understood the task instructions, each Stroop task was preceded by eight practice trials, during which they were required to achieve an accuracy of at least 90% [24]. The order of the three Stroop tasks was randomized across participants to control for potential sequence effects. After completion of the Stroop tasks, participants were given a self‐paced break. They then completed the emotion recognition task. Finally, participants’ estimated IQ was assessed.

### 2.5. Statistical Analysis

Data analyses were conducted using SPSS version 26.0 and R version 4.3.1.

#### 2.5.1. Behavioral Data

First, error trials (i.e., incorrect responses and omissions) and trials with RTs exceeding ±3 standard deviations (SDs) from the mean RT in each condition were excluded for each participant [50]. In total, 4.20% of the trials were removed. The remaining RTs were analyzed as the dependent variable for each Stroop task.

Three repeated‐measures analyses of variance (ANOVAs) were conducted, with group (HCs, individuals with subclinical depression, and patients with MDD) as the between‐subjects factor and condition as the within‐subjects factor. The within‐subjects factor had two levels (congruent vs. incongruent) for the color‐word and face‐word Stroop tasks and three levels (neutral, negative, and positive) for the emotion Stroop task. When significant main effects were observed, post hoc *t*‐tests were performed. When interaction effects were significant, a simple main effect test was conducted.

Moreover, Pearson correlations were computed in each group to assess the relationships among RTs and scores of the CTQ‐SF. For patients with MDD, clinical measures (i.e., scores of the HAMD and durations of illness) were also included in the correlation analyses. False discovery rate (FDR) corrections were applied to control for multiple comparisons.

To examine whether the group differences in RTs reflected a potential speed–accuracy trade‐off (SAT), we conducted two additional analyses. First, Pearson correlations between mean RT and accuracy were then examined within each group and task. Second, inverse efficiency scores (IESs) were calculated by dividing the mean RTs for correct trials by the proportion of correct responses, with lower values indicating greater combined speed–accuracy efficiency. Group differences in IES were examined separately for each task using one‐way ANOVA, followed by post hoc comparisons when appropriate. Group differences were further examined with age, estimated IQ, and years of education included as covariates.

#### 2.5.2. Computational Modeling

To better characterize the latent cognitive processes underlying the behavioral performance, two computational models were applied. The DDM was fitted to RTs and response data from the emotion Stroop task, using the R package brms version 2.22.0 [51]. For the color‐word and face‐word Stroop tasks, the DMC was employed, implemented via the R package DMCfun version 4.0.1 [52].

The DDM assumes that individuals make binary decision by gradually accumulating evidence until a decision boundary is reached [29]. Four parameters representing latent cognitive processes were estimated: (1) the drift rate (*v*), reflecting the speed and direction of evidence accumulation; (2) the boundary separation (*a*), representing the amount of information required to make a decision; (3) the starting‐point bias (*z*), indicating response bias toward one decision boundary; and (4) the nondecision time (*t*), capturing the combined duration of processes occurring outside the modeled evidence‐accumulation stage rather than as a process‐pure measure of stimulus encoding or motor execution. For the emotion Stroop task, group and condition were included as predictors of drift rate (*v*). Condition was dummy‐coded, with the neutral condition specified as the reference level, thereby allowing the drift rate to vary across the neutral, negative, and positive conditions. Group was also included as a predictor of boundary separation (*a*), nondecision time (*t*), and starting‐point bias (*z*). To estimate the posterior distributions of these parameters, four Markov Chain Monte Carlo (MCMC) chains of 8000 samples were run, each with the first 500 samples discarded as burn‐in to allow the chain to converge. Chain convergence was assessed using the Gelman–Rubin statistic [53], with values below 1.10, indicating satisfactory convergence. Group‐wise comparisons of model parameters were performed using Bayesian hypothesis testing, and Bayes factors (BFs) were calculated using the Savage–Dickey method [54]. BFs greater than 1 were interpreted as evidence for the experimental hypothesis. Specifically, a BF_10_ between 1 and 3 is considered inconclusive, 3–10 as moderate evidence, 10–30 as strong evidence, and greater than 30 as very strong evidence [55]. In addition, posterior probabilities (*P*_*P*|*D*_) for the hypotheses of interest were reported, with values ≥ 0.95 considered statistically significant.

Based on the DDM framework, the DMC model proposes that the executed response is determined by a single evidence accumulation process that integrates both controlled processes (i.e., task‐relevant information) and automatic processes (i.e., task‐irrelevant information) [56]. Automatic processes are assumed to facilitate controlled processing in congruent trials and interfere with it in incongruent trials. For example, in the color‐word Stroop task, the semantic meaning of the word may delay evidence accumulation for the ink color in incongruent conditions. In the DMC model, the upper and lower boundaries represent correct and incorrect responses, respectively. In line with previous studies [27, 56–58], six parameters were estimated: (1) the peak amplitude of automatic processes (*A*), (2) the time‐to‐peak amplitude of automatic processes (*t*_peak_), (3) drift rate of controlled processes (*μ_c_*), (4) the decision boundary (*b*), and (5–6) the mean (*R*_mean_), and SD (*R*_SD_) of the nondecision time. Model fitting was performed using the differential evolution optimization algorithm [59], implemented via the R package DEoptim version 2.2‐8 [60] provided by the DMCfun, with the objective of minimizing root mean squared error (RMSE). The resulting DMC parameters were analyzed using multivariate ANOVA (MANOVA) to examine group differences, followed by Bayesian tests to provide complementary evidence for these effects.

## 3. Results

### 3.1. Demographics and Self‐Report

Table 1 presents the demographic information and self‐report scale scores for all participants. Groups did not differ on age or gender distribution. However, patients with MDD had significantly lower education duration and estimated IQ levels than those of HCs. As expected, patients with MDD and individuals with subclinical depression showed significantly higher levels of depressive symptoms and childhood trauma compared to HCs.

**Table 1:** Sample characteristics.

	HC (*n* = 36)	Sub (*n* = 30)	MDD (*n* = 33)	Statistics			
	*M* (SD)	*M* (SD)	*M* (SD)	*F/χ* ^2^	*p*	Partial *η* ^2^	Comparison
**Demographics**							
Age	20.83 (1.99)	20.89 (1.75)	21.30 (3.07)	0.396	0.674	—	—
Age range	19–29	19–27	18–29	—	—	—	—
Gender (male/female)	18/18	11/19	11/22	2.236	0.327	—	—
Education duration	14.22 (1.44)	14.57 (1.72)	13.27 (1.97)	4.909	0.009^∗∗^	—	MDD < Sub = HC
IQ	116.89 (7.66)	113.37 (8.24)	111.36 (11.50)	3.151	0.047 ^∗^	—	MDD < HC

**Self-report scale score**							
BDI	4.33 (4.57)	24.30 (8.11)	20.85 (11.84)	52.169	0.000^∗∗∗^	0.521	MDD = Sub > HC
CTQ_EN	10.19 (3.98)	13.00 (4.73)	13.97 (4.61)	6.790	0.002^∗∗^	0.124	MDD = Sub > HC
CTQ_EA	6.61 (1.84)	9.20 (3.46)	10.03 (4.36)	9.861	0.000^∗∗∗^	0.170	MDD = Sub > HC
CTQ_PN	7.25 (3.33)	8.73 (3.24)	9.65 (3.31)	4.659	0.012 ^∗^	0.088	MDD > HC
CTQ_PA	5.47 (1.00)	5.80 (1.32)	6.88 (2.57)	5.902	0.004^∗∗^	0.109	MDD > Sub = HC
CTQ_SA	5.64 (1.61)	5.57 (1.07)	6.42 (2.98)	1.744	0.180	0.035	—
CTQ_total	44.31 (7.18)	50.17 (8.20)	54.27 (11.64)	10.297	0.000^∗∗∗^	0.177	MDD = Sub > HC

Abbreviations: BDI, Beck Depression Inventory; CTQ, Childhood Trauma Questionnaire; EA, emotional abuse; EN, emotional neglect; HCs, healthy controls; MDD, patients with major depressive disorder; PA, physical abuse; PN, physical neglect; SA, sexual abuse; Sub, individuals with subclinical depression.

^∗^Indicates *p* < 0.05.

^∗∗^Indicates *p* < 0.01.

^∗∗∗^Indicates *p* < 0.001.

### 3.2. Behavioral Performance

Behavioral performance is presented in Table 2 and Figure 2. In the color‐word Stroop task, the group difference was marginally significant (*F* (2, 96) = 2.81, *p* = 0.065, *η_p_* ^2^ = 0.055), with post hoc comparisons suggesting that patients with MDD exhibited longer RTs than individuals with subclinical depression (*p* = 0.073). A significant main effect of group was found in the emotion Stroop task (*F* (2, 96) = 3.16, *p* = 0.047, *η_p_* ^2^ = 0.062) and the face‐word Stroop task (*F* (2, 96) = 6.68, *p* = 0.002, *η_p_* ^2^ = 0.122). Post hoc tests revealed that patients with MDD exhibited significantly longer RTs than individuals with subclinical depression in both tasks (emotion Stroop task: *p* = 0.041; face‐word Stroop task: *p* = 0.001). A main effect of the condition was observed in both color‐word and face‐word Stroop tasks (see supporting results). No other significant main effects or interactions were found across tasks. In addition, the three groups did not significantly differ in the emotion recognition task (*F* (2, 96) = 0.397, *p* = 0.673).

**Figure 2:**
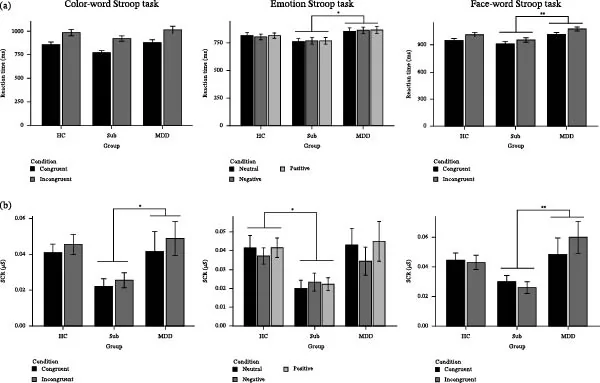
Group comparisons among the three Stroop tasks. (a) Reaction times (RTs) across groups and conditions for the three tasks. (b) Skin response conductance (SCR) amplitudes across groups and conditions for the three tasks. Error bars refer to the standard error computed across participants. HCs, healthy controls; Sub, individuals with subclinical depression; MDD, patients with major depressive disorder.  ^∗^ Indicates *p* < 0.05;  ^∗∗^ indicates *p* < 0.01.

**Table 2:** Group average of reaction time in the color‐word Stroop task, emotion Stroop task, and face‐word Stroop task.

	HC (*n* = 36)	Sub (*n* = 30)	MDD (*n* = 33)
	*M* (SD)	*M* (SD)	*M* (SD)
**Color-word Stroop task**			
Congruent	855.03 (172.83)	770.30 (128.44)	877.33 (176.88)
Incongruent	984.27 (196.71)	919.79 (155.13)	1012.66 (222.96)
**Emotion Stroop task**			
Neutral	816.87 (157.58)	762.14 (152.06)	854.42 (190.43)
Negative	804.57 (147.08)	768.87 (160.55)	865.57 (163.94)
Positive	816.89 (139.10)	769.80 (165.03)	867.87 (174.58)
**Face-word Stroop task**			
Congruent	949.47 (124.67)	908.70 (157.56)	1014.46 (127.66)
Incongruent	1013.06 (138.86)	954.12 (135.79)	1076.33 (128.71)

Abbreviations: HCs, healthy controls; MDD, patients with major depressive disorder; Sub, individuals with subclinical depression.

As for the relationships between behavioral performance and self‐report scale scores, significant associations were found only in the face‐word Stroop task after FDR correction (Figure 3). Specifically, RTs in the incongruent condition were negatively correlated with EN (*r* = −0.68, *p* = 0.014) and total score of childhood trauma (*r* = −0.65, *p* = 0.016) in individuals with subclinical depression.

**Figure 3:**
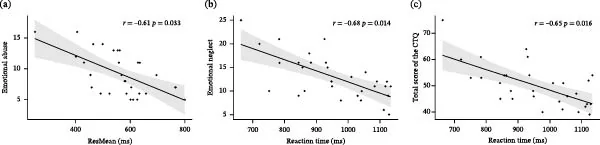
Significant relationship of behavioral results, model parameters, and childhood trauma after FDR correction. (a) Significant negative correlations between scores of emotion abuse and the mean of the nondecision time in color‐word Stroop task. (b) Significant negative correlations between scores of emotion neglect and the mean reaction times in incongruent conditions in face‐word Stroop task. (c) Significant negative correlations between scores of childhood trauma and the mean reaction times in incongruent conditions in face‐word Stroop task. *p*‐Values are FDR‐corrected.

To evaluate the possibility of a SAT, we first examined the correlations between RT and accuracy rate within each group and task. None of the nine correlations was significant (Table S1). Significant group differences on IES were observed for the color‐word Stroop task (*F* (2, 96) = 3.45, *p* = 0.036) and the emotion Stroop task (*F* (2, 96) = 3.60, *p* = 0.031) but not for the face‐word Stroop task (*F* (2, 96) = 1.40, *p* = 0.253) (Table S2). Post hoc comparisons showed that the subclinical depression group had significantly lower IESs than the MDD group in the color‐word (*p* = 0.018) and emotion (*p* = 0.007) Stroop tasks. However, the subclinical depression group did not differ significantly from the HC group in any of the three tasks. After adjustment for age, estimated IQ, and years of education, the group differences remained significant for the emotion Stroop task (*p* = 0.025) and were marginally significant for the color‐word Stroop task (*p* = 0.052).

### 3.3. Computational Modeling

The DMC models provided a reasonable fit to the behavioral data (Figures S1 and S2). To examine group differences in the best‐fitting parameters and mean RMSEs from the DMC models, a MANOVA was conducted (Table S3). In the color‐word Stroop task, significant group differences were found for the parameters *b*, *R*_mean_, and *R*_SD_, which estimated the decision boundary and nondecision components, respectively (*b*: *F* (2, 96) = 3.70, *p* = 0.028, *η_p_* ^2^ = 0.072, BF_10_ = 1.78; *R*_mean_: *F* (2, 96) = 4.31, *p* = 0.016, *η_p_* ^2^ = 0.082, BF_10_ = 2.78; *R*_SD_: *F* (2, 96) = 5.36, *p* = 0.006, *η_p_* ^2^ = 0.100, BF_10_ = 6.41; Figure 4). Post hoc tests indicated that for the separation of decision boundary parameter (*b*), patients with MDD showed a significantly reduced value compared to both individuals with subclinical depression (*p* = 0.047, BF_10_ = 1.05) and HCs (*p* = 0.011, BF_10_ = 3.34). For the nondecision components, individuals with subclinical depression showed a significantly lower *R*_mean_ than patients with MDD (*p* = 0.004, BF_10_ = 8.67) and a lower *R*_SD_ than HCs (*p* = 0.001, BF_10_ = 18.05). In the face‐word Stroop task, marginal significant group differences were found for the *R*_mean_ (*F* (2, 96) = 2.63, *p* = 0.078, *η_p_* ^2^ = 0.052, BF_10_ = 0.75), with individuals with subclinical depression showing lower values than patients with MDD (*p* = 0.025).

**Figure 4:**
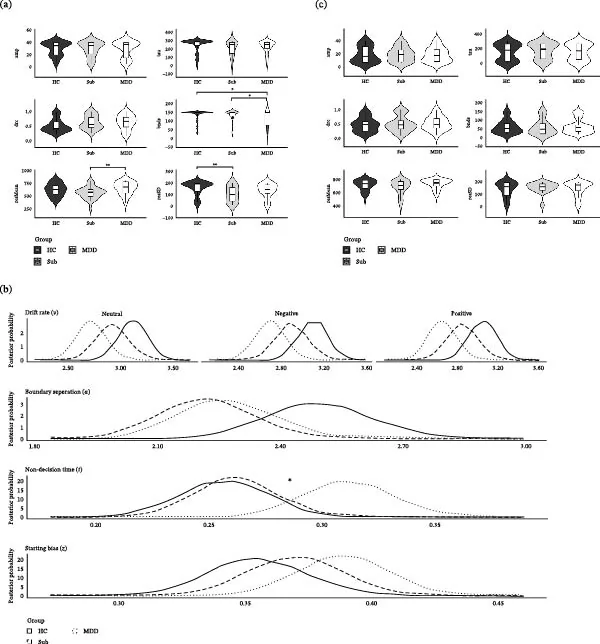
(a) Estimated parameters for the color‐word Stroop task. (b) Posterior distributions of DDM parameters in the emotion Stroop task. (c) Estimated parameters for the face‐word Stroop task. HCs, healthy controls; Sub, individuals with subclinical depression; MDD, patients with major depressive disorder.  ^∗^Indicates *p* < 0.05;  ^∗∗^ indicates *p* < 0.01. Asterisk in the emotion Stroop task indicates < 5% overlap between group‐level posterior distributions, reflecting strong evidence for a difference.

To explore the underlying decision‐making mechanisms during the emotion Stroop task, we fitted a DDM model. Posterior predictive checks indicated that the model provided a good fit to the observed data (Figure S3). The model revealed a significantly prolonged nondecision time in patients with MDD, with a 22% increase relative to HCs (exp (*β*_1_) = 1.22, BF_10_ = 48.18, and *P*_*P*|*D*_ = 0.98) and a 19% increase compared to individuals with subclinical depression (exp (*β*_1_) = 1.19, BF_10_ = 28.41, and *P*_*P*|*D*_ = 0.97). No significant results were observed for the other parameters (Figure 4 and Table S4).

Furthermore, the correlation analyses revealed that the nondecision time parameter (i.e., *R*_mean_) in the color‐word Stroop task was negatively associated with EA after FDR correction (*r* = −0.61, *p* = 0.033; Figure 3).

The significant group differences in behavioral performance and model parameters across Stroop tasks remained unchanged after controlling for demographic covariates (i.e., age, IQ, and educational duration) (Tables S5 and S6). All correlations survived after robust correlation analyses (Table S7).

## 4. Discussion

This study investigated inhibitory control and its underlying computational mechanisms across subclinical depression and MDD using three Stroop tasks, suggesting evidence for a possible inverted U‐shaped trajectory of inhibitory control performance as depressive severity increases. Our key findings were as follows: (a) patients with MDD had significantly longer reaction times than individuals with subclinical depression in both the emotion and face‐word Stroop tasks; (b) DDM and DMC analyses indicated that prolonged nondecision time was the primary determinant of blunted responses in patients with MDD; and (c) in individuals with subclinical depression, childhood trauma, particularly EA and neglect, was associated with faster responses in incongruent face‐word Stroop trials and shorter nondecision time in the color‐word Stroop task.

Across two emotionally salient Stroop tasks, patients with MDD exhibited significantly increased reaction times under affectively loaded conditions (Figure 2). However, no significant differences were observed in the other pairwise comparisons. Some studies have reported that patients with MDD have difficulties to inhibit irrelevant information in both general and emotional‐specific contexts [61–63]. In contrast, several studies have found no significant behavioral differences between individuals with and without depression [23, 24, 64]. These inconsistent results may be partly explained by the heterogeneity of the involved paradigms and samples. By employing three distinct Stroop tasks and recruiting participants spanning subclinical depression through MDD, our study offers a more comprehensive assessment of inhibitory control dysfunction in depression.

Importantly, computational modeling of behavioral data revealed that patients with MDD had a longer nondecision time relative to individuals with subclinical depression (in the color‐word Stroop task) and HCs (in the emotion Stroop task). This finding suggests that nondecisional processes, such as prestimulus encoding and motor response, may be primary contributors to the delayed response patterns observed in MDD. These results align with previous studies showing decision‐making deficits in depression [3, 65]. Our study further extends previous work by indicating that such a delayed response does not stem from the decision‐making process (i.e., drift rate) but instead from extra‐decisional components. Indeed, prolonged nondecision time has been reported in individuals vulnerable to depression [66] and in other populations with mental disorders, such as schizophrenia [67], anxiety disorders [68], and autism spectrum disorders [69]. Moreover, such prolonged nondecision time aligns well with core clinical features of depression, such as lack of motivation [70], diminished concentration [71], and psychomotor retardation [72]. Therefore, by separately estimating distinct decision components, computational models provide us a more nuanced understanding of the mechanisms underlying inhibitory control differences in depression.

Notably, both behavioral and computational findings in the present study converged to suggest a possible inverted U‐shaped pattern across the continuum of depressive severity, whereby reaction times and nondecision time initially decreased and subsequently increased as symptom severity progressed. This nonlinear trajectory may reflect differential engagement of cognitive resources across different stages of depression: While mild to moderate depressive symptoms may promote compensatory cognitive mechanisms, more severe symptomatology may exceed available resources and lead to performance decline. Indeed, one study reported enhanced executive control in minor or moderately depressed individuals [22]. Neuroimaging studies further supported this inverted U‐shaped pattern by demonstrating stage‐dependent neural alterations. For instance, a recent study found increased intrinsic cerebellar activity in individuals with subclinical depression but decreased activity in patients with MDD [73]. Similarly, a task‐based functional magnetic resonance imaging (fMRI) meta‐analysis revealed hyperactivation of the right lenticular nucleus, putamen, and striatum in subclinical depression, in contrast to reduced activation in these regions in MDD [74]. These brain regions are well‐established contributors to motor regulation [75], cognition and emotion control [76], and reward learning [77]. The observed hyperactivation in subclinical depression may thus reflect a neural compensatory response, supporting relatively intact inhibitory control and potentially buffering the progression from subclinical symptoms to full‐blown major depression.

Furthermore, our correlational analyses revealed that the degree of enhanced inhibitory control in individuals with subclinical depression was positively associated with severity of childhood trauma. Specifically, higher levels of EN were related to faster response in the incongruent conditions of the face‐word Stroop task, while EA was associated with shorter nondecision time in the color‐word Stroop task. At first glance, these results seem to diverge from prior research, which has shown the detrimental effects of childhood adversity on cognitive functioning in depression [33, 78, 79]. However, they are not without precedent. Our findings align with the notion of “hidden talents,” which assert that individuals may develop adaptive abilities as a result of their early adversity exposure [35, 80, 81]. For example, Dannehl et al. [36] reported that EA was associated with a better performance of executive function in patients with MDD. Additionally, an fMRI study by Zhong et al. [82] found that healthy individuals with early‐life adversity showed significantly increased activation in key brain regions related to cognitive control and stress regulation, such as the dorsolateral prefrontal cortex, insula, and precuneus, during a psychosocial stress task. Taken together, these findings suggest that childhood maltreatment experiences, particularly EA and neglect, may elicit adaptive hypervigilance and heightened sensitivity to stressors. Such adaptations might facilitate performance on tasks requiring rapid conflict resolution in the short term. However, chronically elevated stress responsivity, mediated via sustained hyperactivation of the hypothalamic–pituitary–adrenal axis, may confer long‐term vulnerability to depression.

Interestingly, we found that patients with MDD exhibited a reduced boundary separation in the color‐word Stroop task compared to both subclinical and HC groups, which contrasts with previous reports of wider decision thresholds in depression [3, 30]. In diffusion models, boundary separation is commonly interpreted as reflecting SAT settings [29], with smaller values indicating that less evidence is required before a response is initiated [83]. In the present study, the reduced boundary separation in patients with MDD, alongside longer RTs, no statistically significant difference in accuracy, and no robust group difference in the controlled drift rate after covariate adjustment, suggests that MDD is characterized by a shift in decision strategy rather than a deficit in information processing. This lower threshold may have partially counterbalanced delays of nondecision processes, as it co‐occurred with significantly prolonged nondecision time. However, this interpretation remains tentative and should be verified in future studies specifically designed to investigate decision‐threshold adjustments in depression.

In contrast to previous studies [13–15], we did not observe an emotional interference in the emotion Stroop task. A similar pattern was observed across the color‐word, emotion, and face‐word Stroop tasks, particularly after controlling covariates. These findings appear to reflect more generalized alterations in task performance rather than effects driven specifically by emotional valence, consistent with the notion of global cognitive impairment in depression [84]. The absence of valence‐specific interference may be related to several methodological and sample‐related factors, including the response modality (e.g., verbal or manual response) [85], the arousal of words [86], the category of emotional words (e.g., general or disorder‐related) [87], and individual differences (e.g., childhood trauma) [88]. Similar nonsignificant valence effects have also been reported in previous studies of depression [89, 90], suggesting that emotional Stroop effects are not always consistently observed across paradigms and samples.

This study has several limitations. First, the interpretation of the nondecision time parameter in DDM needs to be warranted. It has been argued that the standard DDM is limited to separate automatic activation, controlled inhibition, and perceptual encoding [56]. Accordingly, this parameter may reflect a composite of these processes rather than a single distinct cognitive operation. Further decomposition of nondecision time may provide a more precise account of the observed differences in this parameter. Second, our clinical sample primarily consisted of inpatient, and replication in larger and more heterogeneous samples will be essential to strengthen the generalizability of our findings. Finally, the absence of significant associations between depressive severity and inhibitory control in both subclinical and clinical groups may reflect the complex, potentially nonlinear nature of this relationship. Longitudinal studies are suggested to clarify how inhibitory control develops over time and whether it predicts the transition from subclinical depression to MDD.

In conclusion, this study applied three Stroop tasks to comprehensively examine whether inhibitory control alterations in depression reflect a generalized deficit or vary with emotional content. By using the DDM and DMC models, the present study may provide a better characterization of such cognitive control alterations in depression. We found that patients with MDD exhibited prolonged reaction times and nondecision time compared to individuals with subclinical depression, suggesting a possible inverted U‐shaped pattern of inhibitory control across depressive severity. Notably, enhanced inhibitory performance in subclinical depression may reflect a compensatory adaptation, particularly among individuals with higher levels of childhood trauma. These findings highlight the complex interplay between childhood trauma, depression, and cognition.

## Funding

This study was supported by grants from the National Natural Science Foundation of China (Grant 32200906); the Starting Research Fund from Hangzhou Normal University, the STI 2030‐Major Projects (Grant 2021ZD0201705); the Major Project of Philosophy and Social Science Research of the Ministry of Education of China (Grant 22JZD044); the Zhejiang Philosophy and Social Science Laboratory for Research in Early Development and Childcare (Grant RCECDC001); and the Organized Research Program of Hangzhou Normal University.

## Conflicts of Interest

The authors declare no conflicts of interest.

## Supporting Information

Additional supporting information can be found online in the Supporting Information section.

## Supporting information


**Supporting Information** Supporting Information file contains additional information on the experimental tasks, supporting analyses, and results presented in the supporting tables and figures. Table S1: Correlations between reaction time and accuracy rate in each group and task. Table S2: Group comparisons of the inverse efficiency score in the color‐word, emotion, and face‐word Stroop tasks. Table S3: Group average of estimated parameters of DMC in the color‐word Stroop task and face‐word Stroop task. Table S4: Estimates and BFs for the slopes from the DDM in the emotion Stroop task. Table S5: Group comparisons of the reaction time in the color‐word, emotion, and face‐word Stroop tasks (adjusted for age, IQ, and educational duration). Table S6: Group comparisons of the DMC parameters in the color‐word and face‐word Stroop tasks (adjusted for age, IQ, and educational duration). Table S7: Robust correlations between childhood trauma and task performance measures. Figure S1: DMC fits from the color‐word Stroop task and face‐word Stroop task. Figure S2: Parameter recovery for DMC parameters. Figure S3: Convergence check and posterior predictive checking for the DDM. (a) Trace plots illustrating sampling convergence across chains. (b) Posterior predictive checking comparing the observed data (dark blue line) with simulated data generated from posterior distributions (light blue lines).

## Data Availability

Data will be available from the corresponding author upon reasonable request.
