# Supplementary material for: Altered Inhibitory Control Across Subclinical Depression and Major Depressive Disorder: Evidence From Stroop Tasks and Diffusion Modeling
Source: Depress Anxiety. 2026 Oct 25;2026:5711923. doi: 10.1155/da/5711923 (PMC13695802; doi:10.1155/da/5711923)
Supplement: Supplementary file 1 [file DA-2026-5711923-s001.docx]

**Altered inhibitory control across subclinical depression and major depressive disorder: evidence from Stroop tasks and diffusion modeling**

**Supplementary materials**

1. **Supplementary Methods**
2. **Supplementary Results**
3. **Table S1.** Correlations between reaction time and accuracy rate in each group and task.
4. **Table S2.** Group comparisons of the inverse efficiency score in the color-word, emotion, and face-word Stroop tasks.
5. **Table S3.** Group average of estimated parameters of DMC in the color-word Stroop task and face-word Stroop task.
6. **Table S4.** Estimates and BFs for the slopes from the DDM in the emotion Stroop task.
7. **Table S5.** Group comparisons of the reaction time in the color-word, emotion, and face-word Stroop tasks (adjusted for Age, IQ and educational duration).
8. **Table S6.** Group comparisons of the DMC parameters in the color-word and face-word Stroop tasks (adjusted for Age, IQ and educational duration).
9. **Table S7.** Robust correlations between childhood trauma and task performance measures.
10. **Figure S1.** DMC fits from the color-word Stroop task and face-word Stroop task.
11. **Figure S2. Parameter recovery for DMC parameters.**
12. **Figure S3.** Convergence check and posterior predictive checking for the DDM. (a) Trace-plots illustrating sampling convergence across chains; (b) Posterior predictive checking comparing the observed data (dark blue line) with simulated data generated from posterior distributions (light blue lines).

**Supplementary Methods**

**Color-word Stroop Task**

The task comprised of 8 practice trials and 48 formal trials. During the practice, participants had visual feedback mentioning whether the response was correct, incorrect or no response. Participants were required to repeat these practice trials if the accuracy was below 90%. The experiment comprised of 24 congruent and 24 incongruent trials. Participants were required to focus on the fixation in the center of the screen (500 ms), followed by an inter-stimulus interval of 500–1000ms. Then, the target word was shown until the participants answered for a maximum of 2000 ms, followed by a blank of 6000ms. Trials were presented in a random order.

**Emotion Stroop Task**

In this task, color words were replaced with affectively valenced words to produce interference. The task also started with 8 feedback practice trials. Only those whose accuracy was above 90% in practice trials can move into the experimental trials. There were 48 trials experimental trials in which 3 categories words differing in their emotional valences (neutral, negative, and positive) were presented in a block form. Trials within each block were presented in a random order. Example of words include “joyful, fragrant, excellent” for positive (i.e., 欢乐, 芬芳, 优秀 in Chinese, respectively), “upset, awkward, lonely” for negative (i.e., 沮丧, 难堪, 孤独 in Chinese, respectively), “clear, stiff, smooth” for neutral (i.e., 清楚, 坚硬, 光滑 in Chinese, respectively).

**Face-word Stroop Task**

For the face-word Stroop task, participants were shown four types of emotional facial expressions (happy, sad, angry, and disgust), with an emotional word corresponding to one of these four emotions displayed on both cheeks. The word could be either matched (congruent condition) or mismatched (incongruent condition) the facial expression. Participants were instructed to identify the emotion expressed by the face while ignoring the emotional word. Example of Chinese words include “明快,蓬勃”for happy, “苦闷,悲哀”for sad, “愤然,恼火” for anger, “歹毒,刻薄” for disgust.

**Emotion recognition task**

To ensure the participants’ abilities to recognize four types of facial expressions in the face-word Stroop task, an emotion recognition task was employed. In this task, we presented facial expressions with above four emotions (happy, sad, angry, disgust), which included the same and different facial expressions from the face-word Stroop task. The accuracy of each participant was analyzed.

**Robust correlation analyses**

Considering the relatively small sample (n=30) in the subclinical depression group, we further conducted robust correlation methods to confirm the reliability of our results of correlations. First, we conducted bootstrap analyses with 5000 resamples to estimate the 95% confidence interval for the observed correlations. Next, we applied percentage-bend correlation (pbcor), a robust correlation method that reduces the influence of potential outliers while retaining all observations, and that may therefore provide better estimates of the true relationship between the variables (Mair and Wilcox, 2020; Wilcox, 1994). We applied the default bending constant (0.2).

**The parameter-recovery procedure of the DMC**

We conducted a parameter recovery analysis for the DMC applied to the color-word Stroop task. One hundred datasets were simulated using the same proportion of congruent and incongruent trials as in the empirical analysis, and each simulated dataset was fitted using the same estimation procedure and parameter bounds as the observed data.

**Psychophysiological recording and preprocessing**

In addition to behavioral impairments, studies have reported alterations in autonomic activities, such as skin conductance response (SCR) and heart rate variability, during Stroop tasks in both clinical and non-clinical depressed individuals (Bair et al., 2021; Gantiva et al., 2018). Therefore, we also assessed autonomic arousal via SCR amplitudes during each Stroop task.

Electrodermal activity (EDA) was recorded using a Biopac MP160 wireless EDA system (GSR100) and the AcqKnowledge for MP160WS (version 5.0). Prior to data collection, participants were instructed to wash their hands, and the recording sites were prepared using abrasive gel. Then, two disposable electrodes were attached to the palmar surface of the index and middle fingers of the non-dominant hand. Participants were instructed to keep their left hand still throughout the recording to minimize movement artifacts. After completion of the Stroop tasks, the electrodes were removed.

EDA signals were acquired at 1000 Hz, amplified, and low-pass filtered online at a 1-Hz threshold, then resampled to 250 Hz. SCRs were extracted following the standard published guidelines (Braithwaite et al., 2013). Specifically, SCR amplitudes were defined as the base-to-peak differences, with the baseline calculated over the 2-second period preceding stimuli onset and the response window extending from 0.5 to 10 seconds following stimulus onset. To ensure data quality and minimize artifacts and equipment noise, only SCRs with amplitudes greater than 0.02 μS were retained (Measures et al., 2012). To correct for skewness in the distribution of SCR amplitudes, a log-transform was applied. Normality was confirmed using the Kolmogorov-Smirnov test, and subsequent statistical analyses were conducted on the average log-transformed SCR amplitudes. The final sample for autonomic data consisted of 56 participants (patients with MDD: *n* = 10; individuals with subclinical depression: *n* = 19; healthy controls: *n* = 27).

In line with the behavioral data analysis, ANOVAs were also conducted to compare the autonomic responses (i.e., SCR amplitudes) among the three groups for each Stroop task.

**Supplementary Results**

**Behavioral results**

Overall, classic Stroop conflict effects were observed in both the color-word Stroop task (condition: *F* (1, 96) = 214.26, *p* < 0.001, *η*_p_^2^ = 0.691) and the face-word Stroop task (condition: *F* (1, 96) = 27.37, *p* < 0.001, *η*_p_^2^ = 0.222), with participants responding significantly more slowly in the incongruent condition than congruent condition. However, no significant main effect of condition was found in the emotion Stroop task (*η*_p_^2^ = 0.003; adjusted for IQ and educational duration: *η*_p_^2^ = 0.015).

We conducted additional ANCOVA analyses to examine group differences in RT while controlling for the effects of age, IQ, and educational duration. The group effects remain significant in emotion and face-word Stroop tasks (Supplementary Table S3). Post-hoc comparisons indicated that the subclinical depression group showed shorter RTs than the MDD group in the emotion Stroop task, whereas both healthy controls and the subclinical depression group showed shorter RTs than the MDD group in the face-word Stroop task.

**Computational Modeling results**

We also examined the potential confounding effects of age, IQ, and educational duration on DDM or DMC parameters. As for the group comparisons of non-decision time, the overall pattern of results remained unchanged after accounting for above covariates. For the color-word Stroop task, group differences of the parameters *b, R_mean_* and *R_SD_* remained significant (Supplementary Table S4). Post-hoc tests indicated that for the separation of decision boundary parameter (*b*), the MDD group showed significantly reduced values compared to both subclinical depression group (*p* = 0.037) and healthy controls (*p* = 0.015). For the non-decision components, subclinical depression group showed significantly lower *R_mean_* values than the MDD group (*p* = 0.009), as well as lower *R_SD_* values than healthy controls (*p* = 0.001). For the face-word Stroop task, a marginal significant group differences were found for the *R_mean_*, with subclinical depression group showing lower values than MDD group (*p* = 0.026). For the emotion Stroop task, the MDD group remains significantly longer non-decision time than healthy controls (*p* = 0.003) and subclinical depression group (*p* = 0.011).

Only 13 of the 33 patients with MDD were receiving antidepressant medication. Nevertheless, given the potential impact of antidepressant medication on key parameters, we examined Spearman correlations between fluoxetine-equivalent dosage and non-decision time within this medicated subgroup. No correlation reached significant between dosage and non-decision time across Stroop tasks (color-word Stroop task: *ρ* = 0.03, *p* = 0.929; emotion Stroop task: *ρ* = 0.03, *p* = 0.929; face-word Stroop task: *ρ* = 0.51, *p* = 0.075).

**Parameter-correlation results**

The correlations between the generating and recovered values were *r* = 0.714 for boundary separation (*b*) and *r* = 0.700 for controlled drift rate (*μc*), indicating moderate recovery of these parameters under our experimental design (Supplementary Figure S2). Given that recovery was not perfect, we interpreted these parameter estimates cautiously.

**Robust correlation results**

None of the resulting 95% bootstrap confidence intervals included zero, indicating that the observed correlations were stable across resampling iterations. Consistent with the bootstrap results, the percentage-bend correlations remained statistically significant and showed comparable effect sizes across all tested associations (Table S7).

**Automatic results**

Regarding autonomic activity, no significant main effect of condition was found for SCR amplitudes. However, significant group differences emerged in both the color-word Stroop task (*F* (2, 53) = 3.57, *p* = 0.035, *η*_p_^2^ = 0.119) and the face-word Stroop task (*F* (2, 53) = 4.01, *p* = 0.024, *η*_p_^2^ = 0.132), with patients with MDD showing significantly higher SCR amplitudes than individuals with subclinical depression (color-word Stroop task: *p* = 0.016; face-word Stroop task: *p* = 0.007). In the emotion Stroop task, a marginally significant group effect was observed (*F* (2, 53) = 3.00, *p* = 0.058, *η*_p_^2^ = 0.102). Post-hoc comparisons revealed that individuals with subclinical depression had reduced SCR amplitudes compared of healthy controls (*p* = 0.019). No significant relationship was found between SCR amplitudes and self-report scale scores. However, the interpretation of these results needs to be warranted due to the limited sample size of SCR.

**Table S1. Correlations between reaction time and accuracy rate in each group and task.**

|  | Color-word Stroop Task | |  | Emotion Stroop Task | |  | Face-word Stroop Task | |
| --- | --- | --- | --- | --- | --- | --- | --- | --- |
|  | *r* | *p* |  | *r* | *p* |  | *r* | *p* |
| HC | 0.251 | 0.140 |  | 0.14 | 0.414 |  | 0.177 | 0.301 |
| Sub | 0.046 | 0.808 |  | 0.321 | 0.083 |  | -0.088 | 0.643 |
| MDD | 0.092 | 0.610 |  | 0.152 | 0.399 |  | 0.209 | 0.243 |

*Note.* HC, healthy controls; Sub, individuals with subclinical depression; MDD, patients with major depressive disorder.

**Table S2. Group comparisons of the inverse efficiency score in the color-word, emotion, and face-word Stroop tasks.**

|  | HC |  | Sub |  | MDD |  | Statistics | | | |
| --- | --- | --- | --- | --- | --- | --- | --- | --- | --- | --- |
|  | Mean (*SD*) |  | Mean (*SD*) |  | Mean (*SD*) |  | *F* | *p* | Partial *η^2^* | Comparison |
| Color-word Stroop Task | 944 (30.10) |  | 873 (33.0) |  | 993 (31.50) |  | 3.45 | 0.036^*^ | 0.060 | Sub < MDD |
| Emotion Stroop Task | 824 (25.30) |  | 790 (27.70) |  | 890 (26.40) |  | 3.60 | 0.031^*^ | 0.060 | Sub < MDD |
| Face-word Stroop Task | 1225 (34.30) |  | 1177 (37.60) |  | 1264 (35.90) |  | 1.40 | 0.253 | 0.023 |  |

*Note.* HC, healthy controls; Sub, individuals with subclinical depression; MDD, patients with major depressive disorder;

^*^ indicates *p* < 0.05.

**Table S3.** Group average of estimated parameters of DMC in the color-word Stroop task and face-word Stroop task.

|  | **HC (n = 36)** |  | **Sub (n = 30)** |  | **MDD (n = 33)** |  |  | | **Statistics** | | | |
| --- | --- | --- | --- | --- | --- | --- | --- | --- | --- | --- | --- | --- |
|  | ***M (SD)*** |  | ***M (SD)*** |  | ***M (SD)*** |  | ***F*** | ***p*** | | **Partial *η^2^*** | **BF_10_** | **Comparison** |
| **Color-word Stroop task** |  |  |  |  |  |  |  |  | |  |  |  |
| *A* | 30.01 (11.18) |  | 29.40 (12.21) |  | 28.85 (13.38) |  | 0.077 | 0.926 | | 0.002 | 0.10 |  |
| *t*_peak_ | 247.95 (82.72) |  | 214.45 (83.45) |  | 224.96 (78.33) |  | 1.482 | 0.232 | | 0.030 | 0.30 |  |
| *μ*_c_ | 0.52 (0.23) |  | 0.63 (0.22) |  | 0.64 (0.25) |  | 2.766 | 0.068 | | 0.054 | 0.86 | HC < Sub = MDD |
| *b* | 135.21 (29.49) |  | 130.92 (33.26) |  | 112.35 (45.51) |  | 3.698 | 0.028^*^ | | 0.072 | 1.78 | MDD < Sub = HC |
| *R*_mean_ | 621.55 (124.90) |  | 564.58 (117.33) |  | 655.00 (125.95) |  | 4.314 | 0.016^*^ | | 0.082 | 2.78 | Sub < MDD |
| *R*_SD_ | 156.91 (61.11) |  | 105.37 (68.01) |  | 134.03 (62.44) |  | 5.356 | 0.006^**^ | | 0.100 | 6.41 | Sub < HC |
| RMSE | 69.31 (38.82) |  | 65.14 (50.21) |  | 80.13 (68.67) |  | 0.667 | 0.516 | | 0.014 | 0.16 |  |
| **Face-word Stroop task** |  |  |  |  |  |  |  |  | |  |  |  |
| *A* | 18.21 (12.46) |  | 18.39 (11.03) |  | 17.86 (11.08) |  | 0.017 | 0.983 | | 0.000 | 0.09 |  |
| *t*_peak_ | 151.13 (114.28) |  | 171.18 (107.09) |  | 154.03 (110.91) |  | 0.302 | 0.740 | | 0.006 | 0.12 |  |
| *μ*_c_ | 0.47 (0.24) |  | 0.50 (0.27) |  | 0.51 (0.24) |  | 0.289 | 0.750 | | 0.006 | 0.12 |  |
| *b* | 63.49 (39.09) |  | 60.51 (44.31) |  | 64.60 (41.72) |  | 0.080 | 0.923 | | 0.002 | 0.10 |  |
| *R*_mean_ | 712.79 (85.78) |  | 681.50 (114.27) |  | 734.37 (73.43) |  | 2.625 | 0.078 | | 0.052 | 0.75 | Sub < MDD |
| *R*_SD_ | 141.59 (62.49) |  | 153.05 (45.58) |  | 145.12 (61.11) |  | 0.335 | 0.716 | | 0.007 | 0.12 |  |
| RMSE | 148.91 (61.06) |  | 146.28 (54.93) |  | 137.98 (70.87) |  | 0.279 | 0.757 | | 0.006 | 0.12 |  |

*Note*. *A* = the peak amplitude of automatic processes; *t*_peak_ = the time-to-peak amplitude of automatic processes; *μ*_c_ = the drift rate of controlled processes; *b* = the decision boundary; *R*_mean_ = the mean of the non-decision time; *R*_SD_ = the standard deviation of the non-decision time; RMSE, root mean squared error; HC, healthy controls; Sub, individuals with subclinical depression; MDD, major depressive disorder.

^*^ indicates *p* < 0.05; ^**^ indicates *p* < 0.01.

**Table S4.** Estimates and BFs for the slopes from the DDM in the emotion Stroop task.

| **Hypothesis** | **Estimate** | **SE** | **Lower** | **Upper** | **BF_10_** | **Post. Prob** |
| --- | --- | --- | --- | --- | --- | --- |
| dr_GroupSub > 0 | -0.20 | 0.21 | -0.54 | 0.14 | 0.19 | 0.16 |
| dr_GroupMDD > 0 | -0.40 | 0.19 | -0.71 | -0.08 | 0.02 | 0.02 |
| dr_GroupMDD –dr_GroupSub > 0 | -0.20 | 0.21 | -0.54 | 0.15 | 0.21 | 0.17 |
| dr_Conditionnegative > 0 | -0.02 | 0.05 | -0.11 | 0.06 | 0.49 | 0.33 |
| dr_Conditionpositive > 0 | -0.07 | 0.05 | -0.15 | 0.01 | 0.09 | 0.08 |
| dr_Conditionpositive – dr_Conditionnegative > 0 | -0.05 | 0.05 | -0.13 | 0.03 | 0.22 | 0.18 |
| bs_GroupSub > 0 | -0.12 | 0.07 | -0.23 | 0 | 0.06 | 0.06 |
| bs_GroupMDD > 0 | -0.09 | 0.07 | -0.21 | 0.02 | 0.11 | 0.10 |
| bs_GroupMDD - bs_GroupSub > 0 | 0.02 | 0.07 | -0.10 | 0.14 | 1.64 | 0.62 |
| ndt_GroupSub > 0 | 0.02 | 0.10 | -0.14 | 0.19 | 1.39 | 0.58 |
| ndt_GroupMDD > 0 | 0.20 | 0.10 | 0.03 | 0.37 | 48.18 | 0.98 |
| ndt_GroupMDD - ndt_GroupSub > 0 | 0.17 | 0.10 | 0.02 | 0.33 | 28.41 | 0.97 |
| bias_GroupSub > 0 | 0.06 | 0.12 | -0.12 | 0.26 | 2.41 | 0.71 |
| bias_GroupMDD > 0 | 0.15 | 0.12 | -0.05 | 0.34 | 8.52 | 0.90 |
| bias_GroupMDD - bias_GroupSub > 0 | 0.08 | 0.11 | -0.10 | 0.26 | 3.40 | 0.77 |

*Note*. Dr, drift rate; Sub, individuals with subclinical depression; MDD, major depressive disorder; bs, boundary separation; ndt, non-decision time; bias, starting point bias. For each slope (for each line), the first two columns represent the estimated most probable value and its standard error (SE). The ‘Lower’ and ‘Upper’ columns contain the lower and upper bounds of the 95% CI, whereas the ‘Rhat’ column reports the Gelman-Rubin statistic. BF_10_ reports the Bayes factor in favor of the alternative hypothesis; Post.Prob represents posterior probabilities for the interest hypothesis, with a posterior probability of ≥ 0.95 deemed statistically significant.

**Table S5. Group comparisons of the reaction time in the color-word, emotion, and face-word Stroop tasks (adjusted for Age, IQ and educational duration).**

|  | HC |  | Sub |  | MDD |  | Statistics | | | |
| --- | --- | --- | --- | --- | --- | --- | --- | --- | --- | --- |
|  | Mean (*SD*) |  | Mean (*SD*) |  | Mean (*SD*) |  | *F* | *p* | Partial *η^2^* | Comparison |
| Color-word Stroop Task | 919.65 (28.84) |  | 845.04 (31.59) |  | 945.00 (30.12) |  | 2.59 | 0.080 | 0.053 | Sub < MDD |
| Emotion Stroop Task | 812.78 (25.19) |  | 766.93 (28.00) |  | 862.62 (26.31) |  | 3.43 | 0.036^*^ | 0.069 | Sub < MDD |
| Face-word Stroop Task | 981.27 (20.71) |  | 931.41 (22.69) |  | 1045.39 (21.63) |  | 5.76 | 0.004^**^ | 0.110 | HC = Sub <MDD |

*Note.* HC, healthy controls; Sub, individuals with subclinical depression; MDD, patients with major depressive disorder.

^*^ indicates *p* < 0.05; ^**^ indicates *p* < 0.01.

**Table S6. Group comparisons of the DMC parameters in the color-word and face-word Stroop tasks (adjusted for Age, IQ and educational duration).**

|  | Color-word Stroop Task | | | |  | Face-word Stroop Task | | | |
| --- | --- | --- | --- | --- | --- | --- | --- | --- | --- |
|  | *F* | *p* | Partial *η^2^* | Comparison |  | *F* | *p* | Partial *η^2^* | Comparison |
| A | 0.11 | 0.894 | 0.002 |  |  | 0.09 | 0.915 | 0.002 |  |
| t_peak_ | 2.22 | 0.114 | 0.046 |  |  | 0.04 | 0.957 | 0.001 |  |
| μc | 2.12 | 0.126 | 0.044 |  |  | 0.50 | 0.605 | 0.011 |  |
| b | 3.46 | 0.035^*^ | 0.069 | MDD<Sub=HC |  | 0.03 | 0.970 | 0.001 |  |
| R_mean_ | 3.78 | 0.026^*^ | 0.075 | Sub<MDD |  | 2.66 | 0.075 | 0.054 | Sub<MDD |
| R_SD_ | 5.63 | 0.005^**^ | 0.108 | Sub<HC |  | 0.09 | 0.911 | 0.002 |  |

*Note*. A = the peak amplitude of automatic processes; t_peak_ = the time-to-peak amplitude of automatic processes; μc = the drift rate of controlled processes; b = the decision boundary; R_mean_ = the mean of the non-decision time; R_SD_ = the standard deviation of the non-decision time.

^*^ indicates *p* < 0.05; ^**^ indicates *p* < 0.01.

**Table S7. Robust correlations between childhood trauma and task performance measures.**

| **Association** | **Bootstrap 95% CI** | **pbcor** | ***p*** |
| --- | --- | --- | --- |
| CTQ_EA – Stroop_resMean | [-0.779, -0.339] | -0.55 | 0.002^**^ |
| CTQ_EN – Fstroop_incongruent | [-0.855, -0.402] | -0.63 | 0.000^***^ |
| CTQ_total – Fstroop_incongruent | [-0.828, -0.367] | -0.60 | 0.001^**^ |

*Note*. CTQ, Childhood Trauma Questionnaire; EA, emotional abuse; EN, emotional neglect.

^**^ indicates *p* < 0.01; ^***^ indicates *p* < 0.001.

**Supplementary Figures**

**Figure S1.** DMC fits from the color-word Stroop task and face-word Stroop task.

**Figure S2**. Parameter recovery for DMC parameters. We simulated 100 datasets containing 60 trials (50% congruent and 50% incongruent) from a set of random parameters that fell within the ranges of the parameter values obtained in our results. Next, we fitted the model to the simulated data with the exact same procedure as for the experimental data, to examine the reliability of the DMC parameter estimates. The six panels show the Pearson correlations between the generated and the recovered parameter values for the drift rate of controlled processes (drc), the separation between decision boundaries (bnds), the amplitude and time to peak of the automatic processes (amp) (above), time to peak of the automatic processes (tau), the non-decision time (resMean), and the standard deviation of the non-decision time (resSD) (below).

**Figure S3.** Convergence check and posterior predictive checking for the DDM. (a) Trace-plots illustrating sampling convergence across chains; (b) Posterior predictive checking comparing the observed data (dark blue line) with simulated data generated from posterior distributions (light blue lines).

**References**

Bair, A., Marksteiner, J., Falch, R., Ettinger, U., Reyes del Paso, G.A., Duschek, S., 2021. Features of autonomic cardiovascular control during cognition in major depressive disorder. Psychophysiology 58, e13628.

Braithwaite, J.J., Watson, D.G., Jones, R., Rowe, M., 2013. A guide for analysing electrodermal activity (EDA) & skin conductance responses (SCRs) for psychological experiments. Psychophysiology 49, 1017-1034.

Gantiva, C., Araujo, A., Aragão, N., Hewitt, N., 2018. Modulation of physiological responses as indices of attentional bias in Dysphoria. International Journal of Mental Health and Addiction 16, 328-338.

Mair, P., Wilcox, R., 2020. Robust statistical methods in R using the WRS2 package. Behavior Research Methods 52, 464-488. <https://doi.org/10.3758/s13428-019-01246-w>

Measures, S.f.P.R.A.H.C.o.E., Boucsein, W., Fowles, D.C., Grimnes, S., Ben‐Shakhar, G., Roth, W.T., Dawson, M.E., Filion, D.L., 2012. Publication recommendations for electrodermal measurements. Psychophysiology 49, 1017-1034.

Wilcox, R.R., 1994. The percentage bend correlation coefficient. Psychometrika 59, 601-616. <https://doi.org/10.1007/BF02294395>
